# Viral Infection Drives the Regulation of Feeding Behavior Related Genes in *Salmo salar*

**DOI:** 10.3390/ijms222111391

**Published:** 2021-10-21

**Authors:** David Muñoz, Ricardo Fuentes, Beatriz Carnicero, Andrea Aguilar, Nataly Sanhueza, Sergio San-Martin, Cristian Agurto, Andrea Donoso, Leonardo E. Valdivia, Jesús M. Miguez, Lluis Tort, Sebastián Boltana

**Affiliations:** 1 Centro de Biotecnología, Departamento de Oceanografía, Universidad de Concepción, Concepción 4030000, Chile; bio.dnmunoz10@gmail.com (D.M.); b.carniceroarnanz@gmail.com (B.C.); aguilar.espinoza@gmail.com (A.A.); natalysanhueza@udec.cl (N.S.); 2 Departamento de Biología Celular, Facultad de Ciencias Biológicas, Universidad de Concepción, Concepción 4030000, Chile; ricfuentes@udec.cl; 3 Grupo Interdisciplinario en Biotecnología Marina (GIBMAR), Centro de Biotecnología, Departamento de Ciencia y Tecnología de los Alimentos, Facultad de Farmacia, Universidad de Concepción, Concepción 4030000, Chile; sergio.sanmartin.p@gmail.com (S.S.-M.); cagurto@udec.cl (C.A.); andreadonoso@udec.cl (A.D.); 4 Center for Integrative Biology, Faculty of Sciences, Universidad Mayor, Santiago 7500000, Chile; LEONARDO.VALDIVIA@UMAYOR.CL; 5 Centro de Investigación Marina, Laboratorio de Fisiología Animal, Departamento de Bioloxía Funcional e Ciencias de la Salud, Facultad de Biología, Universidade de Vigo, 36310 Vigo, Spain; jmmiguez@uvigo.es; 6 Department of Cell Biology, Physiology and Immunology, Universitat Autonoma de Barcelona, 08193 Barcelona, Spain; Lluis.Tort@uab.cat

**Keywords:** feeding behavior, lipid metabolism, inflammation, infection

## Abstract

The feeding behavior in fish is a complex activity that relies on the ability of the brain to integrate multiple signals to produce appropriate responses in terms of food intake, energy expenditure, and metabolic activity. Upon stress cues including viral infection or mediators such as the proinflammatory cytokines, prostaglandins, and cortisol, both Pomc and Npy/Agrp neurons from the hypothalamus are stimulated, thus triggering a response that controls both energy storage and expenditure. However, how appetite modulators or neuro-immune cues link pathogenesis and energy homeostasis in fish remains poorly understood. Here, we provide the first evidence of a molecular linkage between inflammation and food intake in *Salmon salar*. We show that in vivo viral challenge with infectious pancreatic necrosis virus (IPNV) impacts food consumption by activating anorexic genes such as *mc4r, crf,* and *pomcb* and 5-HT in the brain of *S. salar*. At the molecular level, viral infection induces an overall reduction in lipid content in the liver, favoring the production of AA and EPA associated with the increment of *elovl2* gene. In addition, infection upregulates leptin signaling and inhibits insulin signaling. These changes are accompanied by a robust inflammatory response represented by the increment of Il-1b, Il-6, Tnfa, and Pge2 as well as an increased cortisol level in vivo. Thus, we propose a model in which hypothalamic neurons respond to inflammatory cytokines and stress-related molecules and interact with appetite induction/inhibition. These findings provide evidence of crosstalk between pathogenesis-driven inflammation and hypothalamic–pituitary–adrenocortical axes in stress-induced food intake behavior in fish.

## 1. Introduction

Feeding behavior is a complex activity that is associated with food intake. Fish are excellent experimental models for studying the regulation of feeding behavior because they have versatile feeding habits and patterns. Many inputs regulate the feeding behavior in the brain including homeostatic signals such as body fat stores, food intake, and circulating glucose and fatty acids, and also by non-homeostatic signals such as learning and experience, hedonics, stress, social situation, and opportunity [1]. As in mammals, the hypothalamus of fish receives and processes information from endocrine signals originating in peripheral tissues, precisely from the gastrointestinal tract, liver, adipose tissue, and brain through neuroendocrine actions [2,3].

In the brain, the feeding behavior control resides in the arcuate nucleus of the hypothalamus (ARH), where the pro-opiomelanocortin (Pomc) neurons (anorexigenic) and the neuropeptide Y (Npy)/agouti-related peptide (Agrp) neurons (orexigenic) suppress or increase the appetite, respectively [4]. The Pomc neurons can respond to serotonin (5-HT), dopamine (DA), leptin, and insulin signaling molecules by expressing receptors for each of them [5]. Remarkably, leptin is known to inhibit feeding behavior [6] and exerts control on hypothalamic neurons including Pomc and Agrp neurons [7]. The Pomc neurons initiate a cascade of signaling events under the action of leptin including activation of the signal transducer and activator of transcription 3 (Stat3). This activation drives the expression of the *pomc* gene, triggering the secretion of the anorexigenic Pomc-derived peptide α-melanocyte-stimulating hormone (α-Msh) [8]. Thus, Pomc neurons through α-Msh inhibit the release of orexigenic signals from Npy/Agrp neurons [9].

Alternatively, among the multiple external factors that stimulate orexigenic neurons, it has been described that only the peptide hormone ghrelin exerts an orexigenic effect [3,10,11]. Mechanistically, ghrelin binds to the growth hormone secretagogue receptor (Ghsr) expressed in the brain and Npy/Agrp neurons. This interaction triggers the release of orexigenic peptides such as Npy/Agrp and GABA, two inhibitory neurotransmitters that suppress the anorexigenic Pomc neurons [12,13].

Under stress conditions, Pomc and Agrp display changes in the feeding behavior, mainly because the nutrient-sensing mechanisms and the neural appetite signals do not operate normally [4]. This disturbance modifies the expression of appetite-related neuropeptides [14] to restore internal homeostasis after an external disturbance, mainly by mobilizing fuel stores to make energy available for increased metabolic demand [15]. Among stressors, pathogen infection represents a fundamental challenge for homeostasis maintenance since it involves activating the immune system, resolving the challenge, and protecting the host against potentially toxic inflammatory processes. Upon infection, the immune system triggers the release of immune mediators including pro-inflammatory cytokines such as tumor necrosis factor-alpha (Tnfa), interleukin-1 (Il-1), interleukin-6 (Il-6), and the type I interferons (Ifn-a/b) [16,17].

Pro-inflammatory cytokines activate the transcription factor Stat3, which triggers the production of cyclooxygenase 2 (Cox-2), the enzyme involved in the synthesis of prostaglandin E2 (Pge2) and D2 (Pgd2). Thus, the biogenesis of Pge2/Pgd2 regulates a mechanism that acts in synergy with the central nervous system (CNS) in response to infection, thus inducing the development of fever in mammals, and behavioral fever in fish [18,19]. The polyunsaturated fatty acids such as arachidonic acid (AA) and eicosapentaenoic acid (EPA) are intimately involved in synthesizing proinflammatory and-anti-inflammatory mediators. Specifically, AA is a precursor of Pge2, and EPA can suppress the inflammatory response via Tnfa and Il-6 [20]. A proposed mechanism for this interaction assumes a competition between AA and EPA for cyclooxygenases (Cox), key enzymes involved in prostaglandin biosynthesis [21]. The released cytokines, contributing to the progression of the immune response to pathogen infection, can also activate the HPI axis and induce the secretion of adrenal glucocorticoids such as cortisol [22,23,24]. The release of cortisol triggers the mobilization of energy reserves through glycolysis and gluconeogenesis [25,26]. This mechanism allows the organisms to meet the energetic requirements during stressful scenarios such as pathogenic challenge [27,28]. In this context, cortisol plays a crucial role in regulating the feeding behavior by stimulating the anorexigenic pathway [29]. Thus, the CNS senses signals of stress condition (i.e., cortisol) and triggers the release of corticotropin-releasing factor (Crf) from the hypothalamus [30,31]. This mechanism promotes adrenocorticotropic hormone (Acth) synthesis, which spatially controls food intake in Pomc neurons [8,32,33].

Under stress conditions, the collective data show that in fish, feeding behavior and nutrient-sensing are inhibited [34,35,36]. However, how this system copes with pathogen infections are still poorly understood. Remarkably, to date, the complete understanding of the monoamine release and the neurotransmitters modulating this circuit remains elusive. In the present study, we identified new factors controlling food intake during pathogenesis. We took advantage of the salmon since it is an ectotherm, mobile organism susceptible to infectious pancreatic necrosis virus (IPNV). Previous studies have shown that fish challenged with IPNV induce an inflammatory response within 24 h post-viral challenge [19,37]. In the present study, we show that viral infection impact feeding behavior in fish. This virus-mediated feeding behavior acts in concert with inflammatory cytokine signaling to modulate the activity of orexigenic molecules such as AgRP and NPY. We also assessed the impact of a pathogenic challenge on food intake-related genes and the crucial modulation of the inflammatory response induced by changes in the fatty acid composition. We hypothesized that these changes disrupt the synthesis of lipid-derived inflammatory cues, affecting the immune response of the fish exposed to viral challenge.

## 2. Results

### 2.1. IPNv Viral Infection Increases the Levels of Cortisol

**Figure 1:**
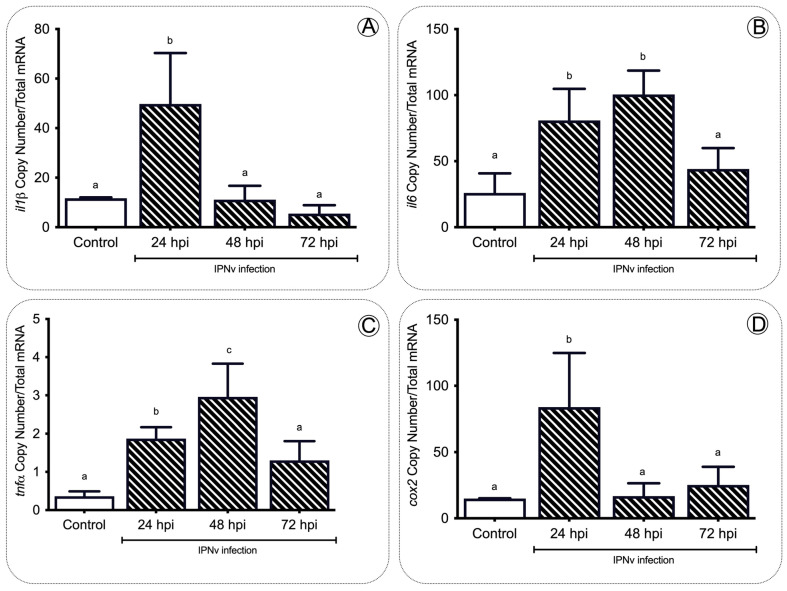
Expression profile of proinflammatory cytokines during infection. The *tnfa* (**A**), *il1b* (**B**), *il6* (**C**), and *cox2* (**D**) gene expression after IPNv infection in the hind kidney of salmon. Control sample corresponds to unchallenged fish. Data represent the mean ± SEM. One-way ANOVA; *p* < 0.05 was considered statistically significant. Different letters denote significant differences.

To address whether food intake behavior is modified by a stress condition, we exposed salmon parr to an IPNV challenge. All fish were infected at 12 °C for 96 h (enough time for the virus to initiate the infection and trigger an inflammatory response, see Boltana et al., 2018 [18,19,37]). We also evaluated IPNV load over time in infected individuals. Quantitative analysis of the VP2 segment mRNA as a readout of viral load (Figure S1) revealed that all fish significantly decreased systemic replication of the virus. Phenotypic analysis performed on each infected fish showed no physical abnormalities. To describe the components of the inflammatory response in salmon, we evaluated the expression levels of *il-β, il-6, tnfa*, and *cox2* genes in the hind kidney by quantitative real-time PCR (Figure 1). The transcripts of proinflammatory cytokines (*il-1β, il-6, tnfa*) and *cox2* were significantly (*p* < 0.05) upregulated at 24 h post-infection (hpi) in IPNv-infected fish (Figure 1A–D). These levels return to baseline at 72 hpi, remaining similar to the control (one-way ANOVA, *p* < 0.05).

**Figure 2:**
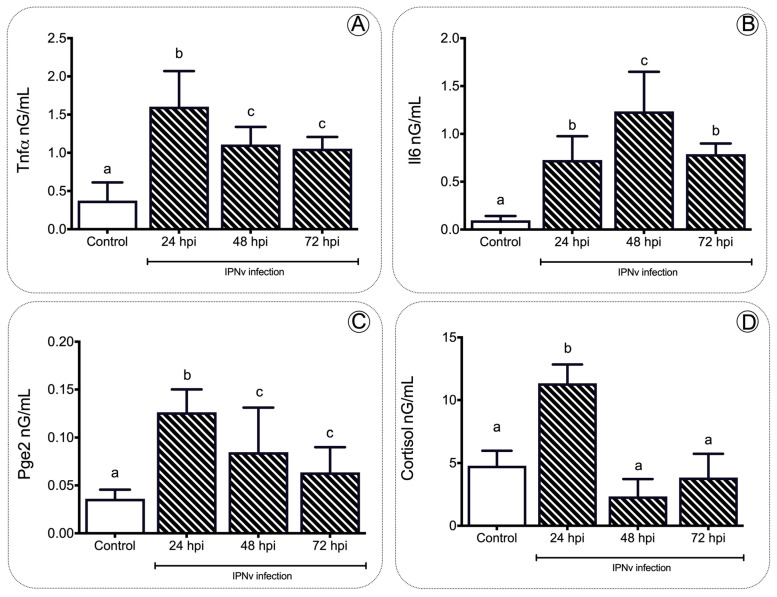
Expression profile of inflammatory mediators during infection. Plasmatic concentration of Tnfa (**A**), Il6 (**B**), Pge2 (**C**) and cortisol (**D**) in salmon after IPNv infection. Data represent the mean ± SEM. One-way ANOVA. *p* < 0.05 was considered statistically significant. Different letters denote significant differences.

To gain insights into the relationship between the stress and inflammation induced by infection viral, we measured the plasma levels of Tnfa, Il-6, Pge2, and cortisol through the ELISA assay (Figure 2). We detected a significant increment in the proinflammatory cytokines Tnfa and Il-6 at 24, 48, and 72 hpi compared to control individuals (Figure 2A,B). Similarly, the plasma levels of Pge2 in the challenged specimens peaked at 24 hpi, reaching 0.1499 ng/mL, showing significant differences (*p* < 0.01) (Figure 2C). This content dropped over time, still being significantly higher than the control group levels. In the inflammation process, the plasma cortisol concentration also showed a significant increase (*p* < 0.001) after viral challenge when compared to the non-challenged group, peaking at 24 hpi with 13.05 ng/mL and returning to baseline levels by 48 hpi and 72 hpi, respectively (one-way ANOVA, *p* < 0.001, Figure 2D).

### 2.2. Upregulation of elovl2 in the Liver of Juvenile Salmon Is Linked with the Increases of AA and EPA during Infection with IPNv

**Figure 3:**
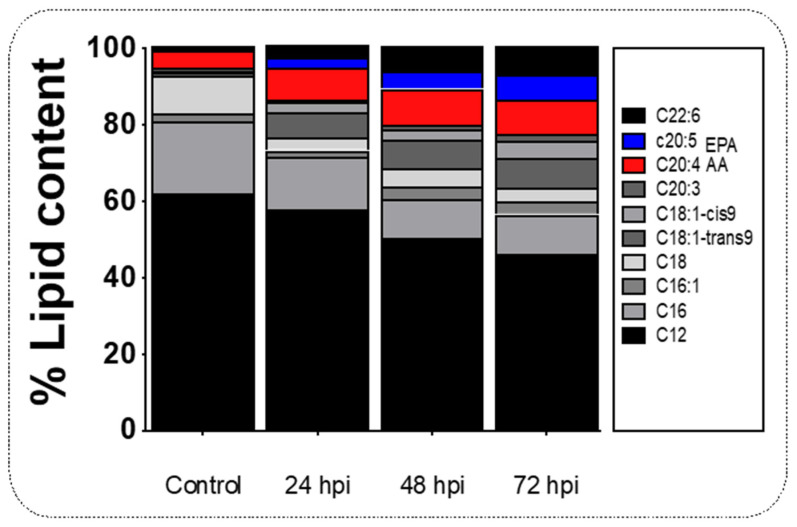
Lipid content profile in the liver tissue of salmon during infection. Composition of lipids in the liver of salmon infected at 24 to 72 hpi. Control sample corresponds to unchallenged fish. Data represent the mean ± SEM. Two-way ANOVA; *p* < 0.05 was considered statistically significant.

Arachidonic acid (AA) and eicosapentaenoic acid (EPA) are two long-chain polyunsaturated fatty acids synthesizing pro and anti-inflammatory cytokines, respectively. We tested how the lipid metabolism in the liver may promote Pge2-mediated inflammatory activity in our infection model by gas chromatography analysis (Figure 3). We found that AA (C20:4) was upregulated at 24 hpi (9.5%), 48 hpi (8.81%), and 72 hpi (7.98%) of infected specimens (Figure 3), which is consistent with increased Pge2 levels at similar time points post-viral challenge (see Figure 2A). Although in the control group EPA (C20:5) was not detected, a 4.78% increase at 24 hpi, 6.7 % increase at 48 hpi, and 4.5% decrease at 72 hpi (Figure 3; *p* < 0.05) was noted. Values for the contents of various lipids present in the control and experimental livers are shown in Table 1.

**Figure 4:**
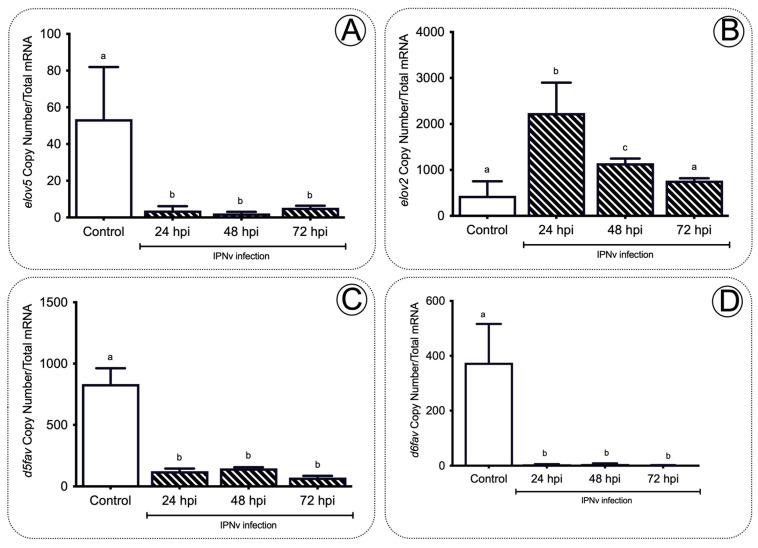
Elongases and desaturases expression profiles in the liver of salmon during infection. The *elovl5* (**A**)*, elovl2* (**B**), *d5fad* (**C**), and *d6fad* (**D**) gene expression profiles after IPNv infection on salmon liver tissue. Control sample corresponds to unchallenged fish. Data represent the mean ± SEM. One-way ANOVA; *p* < 0.05 was considered statistically significant. Different letters denote significant differences.

Long-chain fatty acid biosynthesis depends on the complementary roles of fatty acyl elongases (Elovls) and desaturases (fads). To investigate whether the accumulation of AA and EPA in the liver was accompanied by changes in both lipid elongation and desaturation cycles, we measured mRNA levels of *elovl5* and *elovl2* and *d5fad* and *d6fad*, and two recognized elongases and desaturases codifying genes, respectively. As shown in Figure 4A, *elovl5* liver levels decreased significantly from 57 to three copy numbers after IPNV infection compared to non-infected fish (Figure 4A; *p* < 0.01). Similarly, *d5fad* and *d6fad* transcripts significantly decreased at all post-infection stages (Figure 4C,D; *p* < 0.001). In contrast, *elovl2* was upregulated in infected fish livers at 24 and 48 hpi (Figure 4B; *p* < 0.05).

### 2.3. Anorexigenic Genes Are Upregulated during Infection in Juvenile Salmon

**Figure 5:**
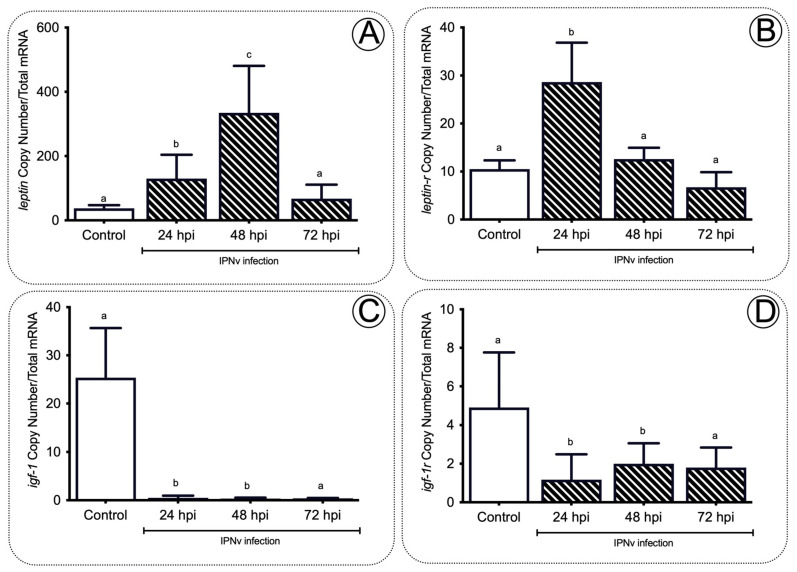
Leptin and insulin expression profiles in the liver of salmon during infection. The *leptin* (**A**), *lepr* (**B**), *igf-1* (**C**), and *igf-1r* (**D**) gene expression after IPNv infection on salmon liver tissue. Control sample corresponds to unchallenged fish. Data represent the mean ± SEM. One-way ANOVA; *p* < 0.05 was considered statistically significant. Different letters denote significant differences.

We assessed the expression levels of several genes related to feeding behavior including expression levels of leptin and leptin receptor (*lepr*). We observed that leptin expression significantly increased (*p* < 0.01) in livers of IPNv challenged fish at 24 and 48 hpi when compared to non-infected individuals, peaking at 48 hpi with 384 transcripts and returning to base levels at 72 hpi (Figure 5A). Leptin receptor (*lepr*) expression also showed a significant increase compared to non-challenged fish (Figure 5B), registering the highest expression levels at 24 hpi with 29 transcripts (*p* < 0.01). To further link metabolic responses and inflammation, we assessed insulin signaling related genes, observing that viral infection significantly decreased transcriptional levels of *igf1* and its receptor *igf1r* in the liver of IPNv challenged fish (Figure 5C,D).

**Figure 6:**
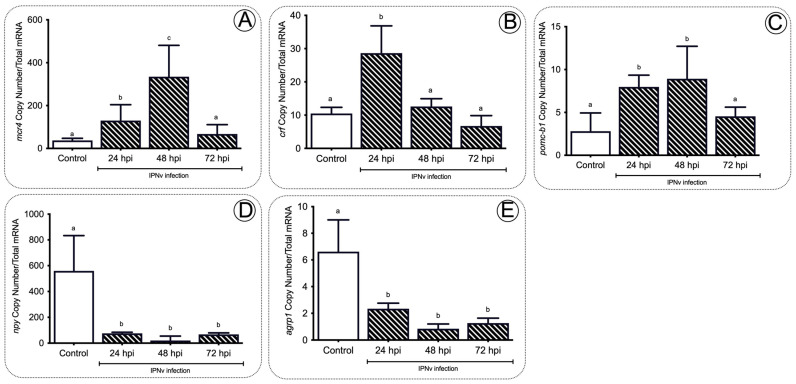
Anorexic and orexigenic gene expression profiles in the brain of salmon during infection. The *mc4r* (**A**), *crf* (**B**), *pomcb* (**C**), *npy* (**D**), and *agrp1* (**E**) gene expression after IPNv infection on salmon brain tissue. Control sample corresponded to unchallenged fish. Data represent the mean ± SEM. One-way ANOVA; *p* < 0.05 was considered statistically significant. Different letters denote significant differences.

As orexigenic and anorexigenic hormone actions represent neural molecular mechanisms regulating feeding behavior, we also explored the mechanism and expression of different orexigenic (*npy, agrp1*) and anorexigenic (*mc4r, crf,* and *pomcb*) related genes within the hypothalamus of IPNv infected fish, analyzing the relationship between an infection challenge and markers for food intake activity. Compared to non-challenged fish, we observed a significant increase in the expression of anorexigenic genes *mc4r, crf*, and *pomcb* (Figure 6A–C) from 24 to 48 hpi in the IPNv challenged individuals. The expression peaks were registered at 48 hpi for *mc4r* (transcript copy number, *p* < 0.05), and 24 hpi for *crf* (transcript copy number, *p* < 0.05) and *pomcb* (transcript copy number, *p* < 0.01). The three gene transcript copy numbers returned to basal levels at 72 hpi. On the other hand, a significantly lower expression of orexigenic genes *npy* (*p* < 0.01) and *agrp1* (*p* < 0.05) was observed in infected individuals when compared to non-challenged fish from 24 hpi onward (Figure 6D,E).

**Figure 7:**
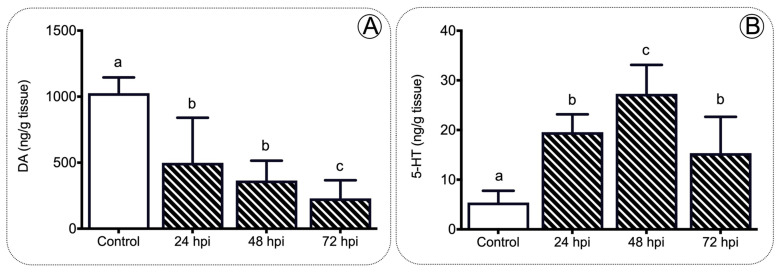
Monoamine content analysis in the brain of salmon. DA (**A**) and 5-HT (**B**) concentration detected in the brain of salmon after IPNv infection. Control sample corresponds to unchallenged fish. Data represent the mean ± SEM. One-way ANOVA; *p* < 0.05 was considered statistically significant. Different letters denote significant differences.

In addition, we analyzed the content of monoamines in the brain during infection related to the anorexigenic neurotransmitters. We assessed dopamine (DA) and serotonin (5-HT) levels upon IPNV viral challenge in juvenile salmon (Figure 7A,B). The monoamine profile of salmon brains revealed significant variations in brain DA and 5-HT content in response to the IPNv challenge (Figure 7A,B). Virus challenged individuals showed progressively lower levels of DA at 24, 48, and 72 hpi (Figure 7A; one-way ANOVA; F3,16 = 5.179; *p* < 0.05). In contrast, 5-HT accumulated over time, reaching maximum levels at 48 hpi and dropping at 72 hpi (Figure 7B; one-way ANOVA: F3,16 = 5.445; *p* < 0.01).

## 3. Discussion

### 3.1. Pro-Inflammatory Cytokines Regulate Feeding Behavior

In the present study, we assessed how inflammatory mediators are related to the regulation of food intake in *S. salar* virus infection. The inflammation response is a hallmark of host defense upon pathogenic infection in fish; as expected, we observed a significant increase in the expression of pro-inflammatory cytokines *tnfa, il1b, il6*, and *cox2* upon infection with IPNv (Figure 1). Under virus-infected individuals, we observed an increase in plasmatic levels of Tnfa, Il-6, Pge2, and cortisol (Figure 2). In general, pro-inflammatory cytokines elicit significant physiological effects on feeding behavior in infected individuals. The collective data show that cytokines play a crucial role in the hypothalamus and modulate appetite behavior [38]. Studies in rats suggest that Tnfa reduces food intake in a dose-dependent manner [39]. The addition of other pro-inflammatory cytokines such as Il-1b also induce the reduction in food intake and anorexia in rats [40]. In mice, microinjections with Il-6 interact with the leptin pathway and cause a decrease in food intake. This mechanism is blocked by i-RNA, leading to a rapid increase in the total weight [41]. Previous reports in mammals have also suggested that pro-inflammatory cytokines induce an increase in the leptin mRNA abundance [42,43,44]. These studies are consistent with our findings, showing that the virus induces a significant release of pro-inflammatory cytokines and the upregulation of anorexic adipose hormone leptin and its receptor in the brain of infected fish (Figure 5).

The activation of the HPI axis during immune challenges is established by increased Acth, which triggers the release of glucocorticoids from adrenal cells. Studies in fish such as *Sparus aurata* and *Oncorhynchus mykiss* also show evidence of an interaction between pro-inflammatory mediators, and the activation of the HPI axis has been suggested [27]. Functional studies in mammals showed that the activation of the hypothalamic–pituitary–adrenocortical (HPA axis or HPI in fish) and the release of stress hormones is blocked by the central injection of the Il-1b receptor antagonist, and suggests the role of pro-inflammatory cytokines in the activation of the HPA axis. Specifically, pathogenic challenges in salmon induce the release of pro-inflammatory cytokines Il-1b, promoting the activation of the HPI axis, and the release of cortisol [45]. In fish, the cortisol increases hepatic leptin mRNA abundance in rainbow trout (*O. mykiss*), both in vivo and in vitro [46]. These results agree with the high cortisol concentrations (Figure 2) and the rise in the mRNA abundance of leptin observed in this study (Figure 5). Thus, the present results suggest that in fish, as in mammals, there is tight crosstalk between the inflammatory activity, activation of the HPI axis (release of cortisol), and the regulation of feeding behavior under pathogenic challenge.

### 3.2. The Gene elovl2 Is Upregulated during Inflammation to Promote the Accumulation of EPA in the Liver of Challenged Salmon

In fish, as in mammals, the responses to pathogenic infections alter the energetic balance of the sick individuals [47]. For example, rodents, birds, and lizards bacteria-challenged displayed decreased locomotory activity, limited growth, and reduced food intake [48,49,50]. In *O. kisutch*, a bacterial challenge also induces reduced fatty acid levels in the liver [51]. In the present study, virus infection induces upregulation of AA and EPA (Figure 3), two polyunsaturated fatty acids directly involved in the synthesis of inflammatory mediators as prostaglandins [20,52]. These observations correlate with the increased mRNA abundance of *cox2* (Figure 1), and the rise in plasmatic Pge2 (Figure 2) observed in virus-challenged individuals.

Our results support the idea that the inflammatory response mediated by plasmatic inflammatory mediators like prostaglandins impacts the liver’s fatty acid composition. In non-infected individuals, the biosynthesis of EPA and AA requires the sequential activity of D6fad and D5fad, respectively [53]. Specifically, Elov2 catalyzes the four responses’ first and rate-limiting reaction that constitutes the long-chain fatty acid elongation cycle. This endoplasmic reticulum-bound enzymatic process allows two carbons to the chain of long- and very-long-chain fatty acids (VLCFAs) per cycle. Elov 5 is also highly involved in the elongation of long-chain polyunsaturated fatty acids such as C18 and C20 PUFA [54]. It has been observed that under virus infection, Elov2, and specifically, Elov5, are highly activated and overregulate lipid metabolism. For example, the human cytomegalovirus (HCMV) infection induces fatty acid (FA) elongation and increases the abundance of lipids with very long-chain FA (VLCFA) tails by the activation of the Elov2 and Elov5 [54]. Specifically, it has been observed that the virus impacts the protein levels of ELOVL5, which elongates PUFAs through a PERK-independent mechanism. These observations show that PERK regulates ELOVL5 during viral infection, creating a balance between the synthesis of lipids with SFA/MUFA tails and PUFA tails. Upon viral infection, we observed a significant decrease in the mRNA abundance of *elov5, d5fad*, and *d6fad* in the liver at all sample times (Figure 4), and an increase in *elovl2* transcripts at 24 and 48 hpi (Figure 4), in stark contrast with the observed levels in control individuals (Figure 4). In this context, the upregulation of *elovl2* promotes the EPA/DHA synthesis pathway to regulate pro-inflammatory cytokines of challenged fish (Figure 4). In the present study, we observed a decrease in plasmatic pro-inflammatory cytokines such as Tnfa, Il-6, and Il-β (Figure 1 and Figure 2), which was correlated with a rise in the EPA content in the liver (Figure 3) and upregulation of *elovl2* mRNA (Figure 4). A previous report showed that EPA suppresses the activity of Nfκb and reduces the production of pro-inflammatory cytokines [55]. Thus, the increment of AA in the liver during challenge would involve a D5fad-independent mechanism in salmon.

### 3.3. A Set of the Anorexic Genes Are Upregulated by Leptin Signaling during Inflammation

In the present study, we observed activation of the anorexigenic pathway after IPNv infection as evidenced by a significant increase in the mRNA abundance of *leptin* and other anorexigenic genes such as *mc4r, crf*, and *pomcb*. Mc4r is a G-protein-linked receptor widely expressed in the hypothalamus and other central nervous system regions and plays a crucial role in energy homeostasis. Specifically, α-Msh binds to Mc4r, inducing an anorexigenic response [56]. Gene for *crf*, whose products have known anorexigenic effects [57], showed the most pronounced and widespread variations of mRNA expression in virally infected fish. As part of the HPI axis, the activated Crf is critical to the release of Acth and cortisol [58]. Thus, our findings agree with previous reports, suggesting that immune challenges actively induce the release of pro-inflammatory cytokines such as Il1, Il6, Tnfa, and Pge2. These cytokines activate the HPI axis by increasing both serum glucocorticoid levels and inducing the hypothalamic mRNA expression of Crf [24], and finally inducing the activation of the anorexigenic pathway.

Insulin plays a crucial role in controlling glucose and energy homeostasis [59], and the intracerebroventricular administration has shown a significant effect of insulin signaling on decreasing appetite in pigs [60]. The regulation of *igf1* and *igf1r* are tightly linked with the activation of feeding behavior and the anorexigenic pathway in mammals and fish [61,62]. The present data showed significant downregulation of *igf1* and *igf1r* in the liver of infected individuals (Figure 5), together with the rise in the mRNA abundance of anorexic genes potentially activated by leptin signaling (Figure 5).

**Figure 8:**
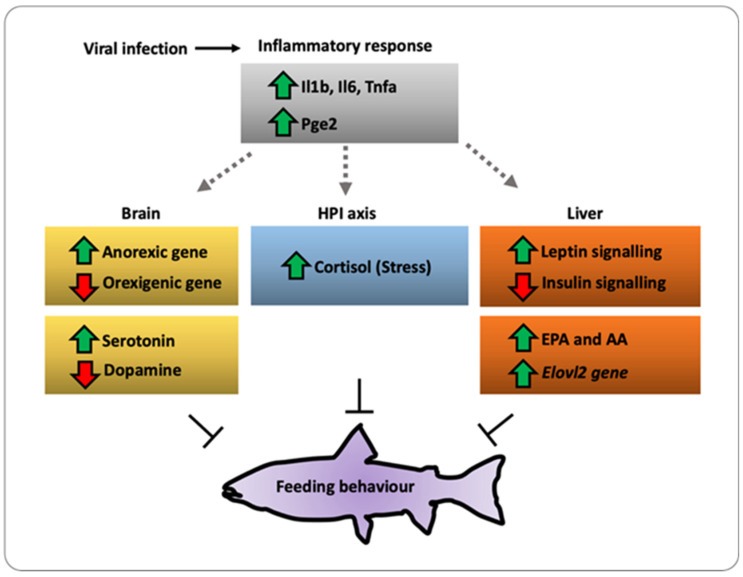
A working model of the immune–sensory interface. Working model based on the results obtained in the present study integrating the HPI axis, immune response, and appetite regulation. We propose that pathogens or organ injury modifies the behavior of critical enzymes such as elov2 and elov5, modifying the lipid metabolism and finally impacting the feeding behavior.

Virus-challenged fish displayed the opposite effects on brain monoamines, increasing serotonin (5-HT) and decreasing dopamine (DA) contents. Several studies have demonstrated an inverse relationship between 5-HT/DA levels and food intake, both in mammals and fish [63,64,65,66,67]. The role of dopamine in feeding behavior is not fully understood in fish, and contradictory results have been reported in different organisms. In mice, stress cues profoundly impact feeding behavior; furthermore, the individuals are hypoactive, apathetic, and aphagic, and finally die because of starvation [68]. In fish, dietary l-dopa (dopamine precursor) induces a decrease in food intake and feed conversion efficiency, affecting growth parameters [69]. Several hormones impact the regulation of the dopamine pathway including leptin and insulin, which directly inhibit dopaminergic neurons [70]. Our findings indicate that DA levels significantly decreased after virus challenge, and it could be related to the high levels of leptin expression observed in the same individuals (Figure 8). Furthermore, the observed increase in brain serotonin levels seems to be related to a higher presence of this neurotransmitter in response to the stress situation resulting from viral challenge, as has been shown in other stress situations in fish. The subsequent action of serotonin on Pomc neurons increasing the hypothalamic release of this peptide [71] would contribute to induce the activation of anorexigenic pathways.

## 4. Materials and Methods

### 4.1. Animal and Hatchery Conditions

All experiments were performed at the ThermoFish Lab, Biotechnology Center, University of Concepcion, Concepcion, Chile, in accordance with international animal research regulations (the British Home Office Regulations, Animal Scientific Procedures Act 1986; care guidelines, EU 2010/63) and following the guidelines for the use of laboratory animals, established by the Chilean National Commission for Scientific and Technological Research (CONICYT), authorized by the Universidad de Concepcion Institutional Animal Care and Use Committee. Juvenile *S. salar* individuals were obtained from AquaGen S.A., Melipeuco, Chile, and maintained on tanks with recirculating freshwater at standard culture conditions.

### 4.2. Infectious Pancreatic Necrosis Virus (IPNv) Challenge

Juveniles *Salmo salar* (121 ± 11.3 mg) were used for the viral challenge (*n* = 80). Fish (*n* = 40) were starved for 12 h and then challenged using the immersion method [37] (in 5 L of water, with a dose of 10 × 10^5^ PFU/mL of clarified supernatant from IPNv-infected CHSE-214 cell monolayers. In parallel, a control group of fish (*n* = 40) was similarly treated by adding 100 mL of virus-free cell culture supernatant to the water. Fish were kept in immersion treatment baths for 2 h and then placed in experimental tanks under constant normothermic (15 °C, constant temperature) conditions. Replicates of 10 fish per treatment (challenge and control) were sampled at 24, 48, and 72 hpi, after being over-anesthetized using MS-222 (Sigma-Aldrich, St. Louis, MO, USA). The control group was obtained for all sampling times (24, 48, and 72hpi). Although no significant differences were observed between the controls over time, only 24 h was selected as a control group for all analysis. Brains, liver, head kidney, and plasma of fish were sampled for each individual, subsequently snap-frozen in liquid nitrogen, and conserved at −80 °C. RT quantitative PCR (RT-qPCR) of each sampled fish was used to estimate the IPNV load by targeting the viral segment virus protein 2 (VP2) using primers WB117 and Universal ProbeLibrary probes (UPL) as previously described [18,19]. All fish used in the experiment were identified as IPNV positive using primers WB117

### 4.3. RNA Extraction, cDNA Synthesis, and Transcript Quantification

All tissue samples were snap-frozen in liquid nitrogen and kept at 80 °C until further analysis. Total RNA was extracted from brain, hind kidney, and liver (100 mg) with the TRI Reagent^®^ (0.5 mL; Sigma-Aldrich) steps, and quantified by absorbance at 260 nm). Only samples with an A260/280 ratio between 1.8 and 2.1, and an A260/230 ratio above 1.8 were used for reverse transcription. Purified RNA integrity was confirmed by agarose-denaturing gel electrophoresis (>9 samples per treatment and time met the suggested quality standards). cDNA was synthesized from 50 μL of total RNA (200 ng/µL) using the RevertAid H Minus First Strand cDNA Synthesis Kit (Fermentas, Waltham, MA, USA), according to the manufacturer’s indications. RT-qPCR was performed using the StepOnePlus™ Real-Time PCR System (Applied Biosystems, Life Technologies, Waltham, MA, USA), and each assay was run in triplicate using the Maxima SYBR Green qPCR Master Mix-2X (Bio-Rad, Hercules, CA, USA). For qPCR assays, 5 μL of synthesized cDNA were diluted with 15 μL of nuclease-free water (Qiagen, Hilden, Germany). Each qPCR mixture contained the SYBR Green Master Mix, 2 μL of diluted cDNA, 500 nmol/L each primer, and RNase-free water to a final volume of 10 μL. The primers used are indicated in Supplementary Table S2 Amplification was performed using the Bio-rad CFX 96 Real Time System on 96-well plates with the following thermal cycling conditions: initial activation for 10 min at 95 °C, followed by 40 cycles of 15 s (s) at 95 °C, 30 s at 60 °C, and 30 s at 72 °C. A dilution series made from known concentrations of plasmids containing the PCR inserts were used to calculate absolute copy numbers for each examined gene in Table S1.

### 4.4. Monoamine Analysis

For the monoamine analysis, brains and whole bodies were snap-frozen in liquid nitrogen and stored at −80 °C. The brain and body content of dopamine (DA) and 5-HT were determined by high performance liquid chromatography with electrochemical detection [72]. The procedure was described previously [73].

### 4.5. Cortisol and Prostaglandin E2 ELISA Assays

Blood plasma was obtained from ten previously sampled fish (see details in Section 4.2) and stored at −80 °C until use. Measurement of plasma cortisol and prostaglandin E2 (Pge2) levels was carried out using a commercial monoclonal enzyme immunoassay (EIA), according to the manufacturer’s instructions (Cayman, MI, USA). This assay has a range from 8.6 to 2000 pg/mL and sensitivity (80% B/B0) of approximately 30 pg/mL. Prior to the determination of Pge2 levels, plasma samples were diluted five times in EIA assay buffer. Results were obtained after absorbance reading at 412 nm. Samples were stored at −80 °C until being used.

### 4.6. Lipid Content

Lipids were extracted from liver (three fish per time and treatment; *n* = 18 total) as described by Bligh and Dyer [74]. Subsequently, transesterification was conducted by adding 2 mL of borontrifluoride (BF3)/methanol (12%) and then incubating at 100 °C for 30 min. After cooling the samples, 1 mL of isooctane was added, followed by stirring. To separate the phases, 2 mL of saturated NaCl was added. The upper phase was transferred into 2 mL amber vials and dried under a stream of nitrogen. Then, it was resuspended in 100 µL of hexane and analyzed on a gas chromatograph, where a mixture of 36 fatty acid methyl esters (FAME) (Restek, Food Industry FAME MIX) was used as the standard. The temperature range to allow chromatography was 100 °C to 240 °C, maintaining the final temperature for 20 min. The injection volume was 2 µL per sample and the carrier gas was nitrogen at 100 kPa. The injector temperature was 225 °C and the FID detector temperature was adjusted up to 250 °C. The data were obtained using the Autochro Data Module interface and Autochro 3000 software (Young Lin Instrument). The FAME profiles of the samples were identified by comparing the retention times and the area of the FAME peaks (mV) with the standard.

### 4.7. Statistical Analysis

The presented data are expressed as mean ± standard deviation (SD). Sigma Plot (version14.0) and GraphPad Prism 7 software were used for statistical analysis and graphing, respectively. The data obtained from gene expression after the absolute quantification of mRNA by real-time polymerase chain reaction (qPCR) were analyzed to determine the normality and homogeneity of the variations using Shapiro–Wilk. When necessary, the data were transformed into log10 to achieve homogeneity and homogenized variances. Data obtained for cortisol and prostaglandin E2 were analyzed by one-way ANOVA, while fatty acids by two-way ANOVA, followed by a post-hoc Tukey HSD test for multiple comparisons. These last tests were also used for monoamine content analysis. All statistical analyses were performed using the SigmaPlot version 14 (SigmaPlot, Stata Statistical Software). Non-parametric statistics were also used when normality tests failed. Means and significance of test groups were compared to the controls. The significance indicator, or letter, above each graph represents a significant difference relative to control. A *p* < 0.05 was considered statistically significant.

## 5. Conclusions

To our knowledge, these novel results identify an unprecedented link between inflammation and regulation of the appetite in fish. These results also correlate with differentially expressed neuropeptides and receptor complexes. We sought to solve a paradigm involving the relation between stress response and appetite-regulating genes in fish subjected to an immune challenge. Such a paradigm consists of the onset of a hypothalamic cue that leads to the execution of an endocrinological response. However, further functional assays are required to fully demonstrate this hypothesis. This effort may include loss-of-function studies of the Pomc or leptin using genome editing technologies in a fish model (SB and DM, unpublished data). For the first time, our findings show that fish leads to a neuro–immune interaction, which might modulate the systemic inflammatory response and the regulation of the appetite under pathogen infection. Our work points to a conserved neural–immune link contributing to a better understanding of the pathogen-mediated inflammatory disease and immune disorder responses during metazoan evolution. In conclusion, this study generates a more complete molecular map of the neuroimmune regulation of the appetite in fish. Our molecular characterization opens the door for further discovery of related gene functions in a complex genetic model.

## Figures and Tables

**Table 1:** Average composition of the fatty acid methyl esters of liver from IPNV infected salmon in each sample time.

LIPID/HPI	Control	24 hpi	48 hpi	72 hpi
Dodecanoic acid C12:0	62 ± 12.12	50.40 ± 9.52	46.31 ± 10.51	62.96 ± 14.23
Hexadecanoic acid, C16:0	19.1 ± 2.90	10.33 ± 3.80	10.31 ± 4.31	11.58 ± 4.12
Palmitoleic acid, C16:1	1.96 ± 0.97	3.14 ± 1.91	3.55 ± 2.01	0.36 ± 0.03
Stearic acid, C18:0	9.82 ± 2.13	5.01 ± 0.88	3.40 ± 1.61	3.55 ± 2.04
Oleic acid, C18:1 trans 9	0.98 ± 0.31	7.40 ± 2.90	7.93 ± 2.86	6.19 ± 2.98
cis-8,11,14-Eicosatrienoic acid methyl ester	0.20 ± 0.01	2.66 ± 1.43	4.53 ± 1.72	1.23 ± 0.91
Methyl eicosatrienoic C20:3	1.01 ± 0.31	1.06 ± 0.47	1.69 ± 0.81	0.11 ± 0.105
Arachidonic acid (AA), C20:4	4.38 ± 2.01	9.50 ± 0.68	8.81 ± 3.65	7.98 ± 3.51
Eicosapentaenoic acid (EPA), C20:5	0.10 ± 0.03	4.78 ± 2.88	6.77 ± 3.06	4.55 ± 1.27
Docosahexaenoic acid (DHA), C22:6	4.43 ± 1.97	5.74 ± 1.91	6.70 ± 2.75	1.50 ± 0.94

Data obtained by HPLC from liver of salmon infected and no infected (control). Numbers represent the lipid content in percentage ± SD.

## Supplementary Materials

The following are available online at https://www.mdpi.com/article/10.3390/ijms222111391/s1.
