# Supplementary material for: Viral Infection Drives the Regulation of Feeding Behavior Related Genes in Salmo salar
Source: Int J Mol Sci. 2021 Oct 21;22(21):11391. doi: 10.3390/ijms222111391 (PMC8583931; doi:10.3390/ijms222111391)
Supplement: Supplementary file 1 [file ijms-22-11391-s001.zip › ijms-1327659-supplementary.pdf]

Supplementary Material

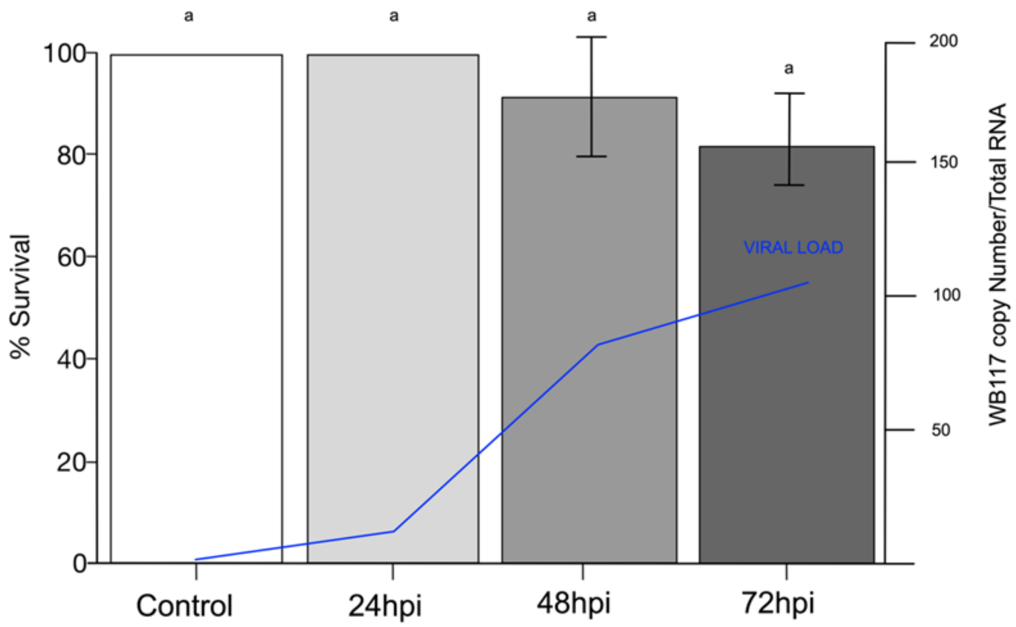

Figure S1. Survival rate and Viral load expressed as the abundance of WB117 segment of the IPNV virus by RT-qPCR in spleen. Bar graph showing differences in mRNA abundances between different times point and control (mean ± s.d., Two-way ANOVA followed by Tukey's post-hoc test). Different letters denote significant differences. Mean and standard error of IPNV copy number per ng of total RNA for the VP2 IPNV viral segment.

Table S1. mRNA primer sequences used for absolute RT-qPCR analysis.

Gene	Name	Sequence Primer 5'-3'	Amplicon Size (bp)	E (%)	Tm (°C)	GenBank
agrp1	Acyl-CoA Retinol O-Fatty-Acyltransferase	F: GCGTTCTCCCCGTCGCTGTA<br>R:TGTTAGGGGCGCCTGTGAGC	156	103	64	XM_014182677.1
npy	Neuropeptide Y	F: CCCCGACACACTGGATACAC<br>R:CATATCTGGACTGTGGGAGCG	77	101	64	NM_001146681.1
mc4r	Melanocortin 4 Receptor	F: CACCGACAACAACACCAGC<br>R:CAGACTAATGAGGCCCAGCG	97	99	62	XM_014127040.1
crf	Corticotropin-Releasing Factor	F: AACCAGCTCGACGACTCGATGG<br>R: GCTATGGGCTTGTTGCTGTAACTG	135	103	60	BT057824
leptin	Leptin	F: GGTGATTAGGATCAATAAGCTGGA<br>R: GAAGAGCATTAGGTCCTGGTAGAA	148	108	60	FJ830677.1
lepr	Leptin Receptor	F: GGGAAGGCTGACACTATGGA<br>R: CTTGTCTGGGACTGACGTAGG	128	98	60	AB489201
pomcb1	Proopiomelanocortin b1	F: CTTCCGCTGGGGCAAACC<br>R: CTCCCGCCATCTTGTCCT	146	101	60	NM_001128604.1
cart	CART Prepropeptide	F: AGTGTTGATTGCCAGAAGCAGATG<br>R: CATGTTGTCTTGCTGCCCGCC	117	106	62	XM_014141510.1
igf1	Insulin Like Growth Factor 1	F: CCTGTTCGCTAAATCTCACTTC<br>R: TACAGCACATCGCACTCTTGA	226	97	60	EF432852
igf1-r	Insulin Like Growth Factor 1 Receptor	F: GGGGCTCTCCTTCTGTCCTA<br>R: AGAGATAGACGACGCCTCCTA	176	111	60	EU861008
elovl2	ELOVL Fatty Acid Elongase 2	F: CGGGTACAAAATGTGCTGGT<br>R: TCTGTTTGCCGATAGCCATT	145	108	64	TC91192
elovl5	ELOVL Fatty Acid Elongase 5	F: ACAAGACAGGAATCTCTTTCAGATTAA<br>R: TCTGGGGTTACTGTGCTATAGTGTAC	137	98	62	AY170327
d5fad	delta-5 fatty acyl desaturase	F: GTGAATGGGGATCCATAGCA<br>R: AAACGAACGGACAACCAGA	192	101	60	AF478472
d6fad	delta-6 fatty acyl desaturase	F: GTGAATGGGGATCCATAGCA<br>R: AAACGAACGGACAACCAGA	192	105	58	GU294488
Tnfa	Tumor Necrosis Factor-Alpha	F: AGGTTGGCTATGGAGGCTGT<br>R: TCTGCTTCAATGTATGGTGGG	250	108	62	NM_001123589.1
Il1b	Interleukin 1 Beta	F: CGTCACATTGCCAACCTCAT<br>R: ACTGTGATGTACTGCTGAAC	200	98	62	AY617117
il-6	Interleukin 6	F: CAGCGGAGAGGTAGTCTGGA<br>R: CGTCCAGAAAGCAGTGGACT	139	107	60	DQ866150
cox-2	Cytochrome C Oxidase II	F: CAGTGCTCCCAGATGCCAAG<br>R: GCGAAGAAGGCGAACATGAG	99	94	60	AY848944.1
